# Integration of former child and adolescent study participants into a national health online panel for a longitudinal study on young adult mental health

**DOI:** 10.1186/s12874-025-02613-2

**Published:** 2025-07-01

**Authors:** Michael Lange, Vera Birgel, Stefan Damerow, Johannes Lemcke, Jennifer Allen, Hans Butschalowsky, Susanna Christen, Caroline Cohrdes

**Affiliations:** 1 Department 2 Epidemiology and Health Monitoring, Robert Koch Institute, Gerichtstraße 27, Berlin, 13347 Germany; 2 German Centre for Mental Health [DZPG], Berlin, Germany

**Keywords:** Longitudinal study, Panel infrastructure, Re-contact, Loss to follow-up, Sample weights, Mental health, Transition, Emerging adulthood, KiGGS, JEPSY

## Abstract

**Background:**

Longitudinal studies are essential for understanding health trajectories and determinants over time. Successfully re-engaging and monitoring participants at key stages such as the transition from adolescence to adulthood is crucial. This stage of life is characterized by many changes and challenges and is considered critical for the manifestation of mental health problems. This study addresses challenges of contacting, re-activation and comparability of individuals in transition to adulthood with the initial population-based sample last contacted up to 9 years ago.

**Methods:**

In 2024, former participants of the “German Health Interview and Examination Survey for Children and Adolescents” (KiGGS) aged 16–25 years were invited to register in the new online panel infrastructure “Health in Germany” and participate in the “Study on Mental Health in Emerging Adulthood” (JEPSY) via a push-to-online panel approach. Logistic regression models identified predictors of registration and study participation. Weighting adjustments were applied to analyse and correct for selective participation. To assess potential selection bias, life satisfaction of JEPSY participants was benchmarked against a representative sample.

**Results:**

Among 11,737 invitees, 4,451 (37.9%) registered, and among these, 3,063 (68.8%) completed the JEPSY survey. For participants aged 11 + years, higher probabilities for registration and participation were evident for females (OR = 2.61 [95% CI: 2.37–2.88]); OR = 2.85, [2.56–3.18]), individuals with higher occupational (OR = 1.08, [1.04–1.13]; OR = 1.06, [1.01–1.11]) or educational status (OR = 1.13, [1.08–1.17]; OR = 1.13, [1.08–1.17]). Individuals with higher emotional problems were less likely to register (OR = 0.98, [0.97–0.99]) and participate (OR = 0.98, [0.97–0.99]). Weighting adjustments reduced biases but increased statistical variance. Benchmarking life satisfaction revealed no significant differences between JEPSY participants and their representative counterparts.

**Conclusions:**

This study demonstrated the feasibility of re-activating former participants and integrating them into a modern online panel. While re-activation was successful for a substantial proportion of invitees, specific challenges (e.g., selective re-participation) remain. The findings provide insights for refining re-contact and retention strategies to improve representativeness and quality of longitudinal mental health research, ultimately enabling more accurate monitoring of health trajectories and their determinants over time.

**Supplementary Information:**

The online version contains supplementary material available at 10.1186/s12874-025-02613-2.

## Contributions to the Literature

- By integrating pre-existing samples into an online panel infrastructure, this study highlighted the potential to re-activate former participants and link past data to current findings for longitudinal analyses.
- Especially for young adults who had previously participated as children or adolescents and were now transitioning from the requirement of parental consent to independent study participation, it was pertinent to investigate the efficacy of this push-to-panel approach.
- This research provided insights into selection effects and participation dropout, contributing to the improvement of future longitudinal research methodologies.

## Background

Longitudinal studies play a crucial role in understanding health trajectories and identifying determinants of health outcomes over time. However, maintaining participant engagement across multiple waves of data collection poses significant challenges, particularly when long periods elapse between contacts. This methods paper focuses on the integration of former participants from the German Health Interview and Examination Survey for Children and Adolescents [Studie zur Gesundheit von Kindern und Jugendlichen in Deutschland, or KiGGS] into the newly established online panel infrastructure “Health in Germany” [Gesundheit in Deutschland], demonstrating how methodological innovations can address these challenges.

KiGGS is a large-scale, nationally representative study conducted by the Robert Koch Institute (RKI) to monitor the health of children and adolescents in Germany. Its combined cross-sectional and longitudinal design, beginning with the baseline survey in 2003–2006, followed by waves 1 and 2, provides comprehensive data on physical and mental health, health behaviours, and social determinants [1]. The RKI Study on Mental Health in Emerging Adulthood [Studie zur psychischen Gesundheit von jungen Erwachsenen in Deutschland, or JEPSY] builds on this foundation by re-activating a subset of participants from KiGGS Wave 2. This transition from adolescence to emerging adulthood offers unique opportunities to investigate how early life conditions are associated with mental health trajectories.

Together, the KiGGS and JEPSY studies provide a case example for addressing a key methodological challenge in longitudinal research: how to re-activate participants after long gaps, particularly when transitioning from parental to individual consent and amidst changes in participants’ lives that may affect engagement. By leveraging the Health in Germany panel infrastructure, this integration demonstrates how former samples can be re-activated within modern research frameworks.

### Challenges in re-contacting and longitudinal investigation of emerging adults

Besides high initial participation, high retention rates across survey waves are important in longitudinal studies, as decreasing participant numbers reduce statistical power and introduce bias, affecting the generalizability of results [2–4]. Avoiding dropout and missing data is especially challenging in the case of adolescents and young adults, who are in a critical phase of life transitions, such as graduating from school, entering vocational training, starting university, or beginning employment [2]. Such transitions often disrupt study participation by complicating the maintenance of up-to-date contact information and reducing participants’ availability or motivation to engage [5].

The issue is further compounded when the final contact occurred a significant period of time before. This was the case with the KiGGS participants, who were last contacted up to 9 years prior. During this period, the participants had transitioned from childhood or adolescence to emerging adulthood. Re-activating these individuals required not only overcoming logistical challenges, such as outdated home addresses, but also addressing potential shifts in motivation. Unlike in previous waves, where participation required parental or legal guardian consent and the child’s willingness — with their formal consent mandatory from the age of 14 —follow-up studies like JEPSY now depend solely on individuals’ willingness to participate.

It may not have been reasonable to assume that the study participants would have the same (high) motivation to participate as their parents did, based on the assumption that parents are more likely to be highly motivated to find out about their child’s health (individual examination results were communicated by letter in KiGGS) or to contribute to improving the health situation by participating in the study, while the adolescents and young adults– as described above– may have had other priorities. This raised additional challenges, as in the KiGGS cohort, older age as well as other sociodemographic characteristics (e.g., male sex, lower family socio-economic status [SES] and the construct of migration background as used in KiGGS^1^) were associated with a lower probability of re-participation [8].

While retention strategies are an important topic in longitudinal studies [3], there are only a few examples that deal with similar re-activation issues, such as (a) re-contacting former longitudinal study participants after a long interruption for a new wave [9], (b) re-contacting former study participants to collect information that only turned out to be relevant after the previous survey [10, 11], or (c) re-contacting participants to obtain consent for secondary data use [12, 13].

### The health in Germany study and panel infrastructure

To enable a faster and more flexible provision of primary data, in 2024 the RKI established the Health in Germany panel infrastructure [14]. This infrastructure consists of a probabilistic sample drawn from population registers to represent the German population aged 16 years and older. A recruitment survey (“welcome survey”) was conducted with the option to register for the panel thereafter. A key strength of this infrastructure is that registered individuals consent to ongoing participation, enabling regular and systematic data collection for robust longitudinal research. Registration and data collection are mainly conducted online, with the option of paper questionnaires available to accommodate different preferences (i.e., mixed-mode design).

Additionally, the panel infrastructure allows for the re-activation and integration of existing samples from the RKI health monitoring system [15, 16]. This capability was utilized for the first time with the transfer of a portion of the KiGGS Wave 2 sample into the panel infrastructure. For this purpose, former participants of KiGGS Wave 2 who were 16 to 25 years old (as of 1 March 2024) were invited to register with the panel infrastructure. This registration not only enabled their participation in the JEPSY study but also laid the groundwork for inviting them to future studies within the panel framework.

For investigating the longitudinal research questions of the JEPSY study, the integration of an existing sample into the panel infrastructure had the advantage that the survey and examination data available from KiGGS could be linked to the newly collected JEPSY data. Furthermore, a panel infrastructure in general simplifies the management of longitudinal studies by enabling contact with individuals via email for study invitations and retention activities, eliminating the need for time-consuming research of home addresses. Additionally, the online mode streamlines data collection by removing the need to print and mail questionnaires. For study participants, access to surveys is also made easier, as they can complete questionnaires on their mobile devices or home computers. Furthermore, participants can conveniently access their incentives and, if preferred, accumulate rewards over time. In the young adult age group, online accessibility is generally assumed as the percentage of internet users is almost 100% [17]. However, encouraging former KiGGS participants to register initially required contacting them by post.

### The German health interview and examination survey for children and adolescents (KiGGS Study)

The KiGGS study is part of RKI’s health monitoring [15, 16] and represents a combined cross-sectional and longitudinal study. The baseline survey, conducted between 2003 and 2006, aimed to assess the health situation of children and adolescents aged 0 to 17 years living in Germany at that time. To ensure that the sample reflected the German population structure, the baseline survey used the population registers as a sampling frame [18]. The sampling procedure comprised two stages: In the first stage, 167 study locations (sampling points) were selected to represent the settlement structure of Germany. In the second stage, the addresses of girls and boys aged 0 to 17 were randomly drawn from local population registers within these sampling points. A total of 17,641 children and adolescents, along with their parents, took part. Two further cross-sectional waves of the survey were conducted in order to collect up-to-date data on the health situation of 0–17-year-olds living in Germany, including KiGGS Wave 1 as a computer-assisted telephone interview survey from 2009 to 2012 [19, 20] and KiGGS Wave 2– a health interview and examination survey from 2014 to 2017 [21]. In addition, participants in the baseline survey who had agreed to be contacted again were still living in Germany and who had not passed away were re-invited to take part in both waves 1 and 2 (KiGGS cohort; [8].

KiGGS covers a broad spectrum of topics, including physical health, mental health, health-related behaviours, use of healthcare services, and living conditions. The survey program, which was conceptualized for six age groups (five age groups under 18 years; one age group 18 + years), comprised questionnaires for parents (of 0–17-year-olds) and for the study participants themselves (from the age of 11). In addition, the baseline survey and part of Wave 2 included physical examinations and laboratory analyses, like blood and urine testing [22, 23].

### Emerging adulthood and mental health

Emerging adulthood, broadly defined as the period between 18 and 25 years, is marked by significant changes in life circumstances, roles, and responsibilities [24]. This transitional phase presents unique challenges, including the shift from paediatric to adult healthcare systems, changes in social networks, and increased demands for autonomy and self-regulation [25]. While these factors provide opportunities for growth, they also increase vulnerability to mental health issues. Evidence suggests that the prevalence of mental health disorders peaks during this life stage, with anxiety, depression, and other conditions being particularly common [26]. The COVID-19 pandemic further exacerbated these issues, with studies reporting rising mental health challenges among adolescents and young adults globally and in Germany [27–29].

Longitudinal cohort studies are essential for understanding dynamic processes that influence mental health during this critical period. By examining factors such as environmental or social influences, healthcare pathways, and individual developmental trajectories, these studies can provide valuable insights for designing targeted prevention and intervention programs [30, 31]. The JEPSY study, conducted as part of the German Centre for National Mental Health [Deutsches Zentrum für Psychische Gesundheit, or DZPG] [32], aimed to address these questions by re-activating KiGGS participants during their transition to adulthood. JEPSY was designed with a focus on mental health, as well as a selection of sociodemographic and social factors.

### The current research

The current study aimed to assess the feasibility of integrating former population-based cross-sectional and cohort participants into a panel infrastructure, with a focus on adolescents transitioning into young adulthood. Therefore, the methodology, including the recruitment process of former KiGGS participants and strategies to enhance re-participation, were described. Moreover, the representativeness of the sample was examined by analysing participation rates, potential selection bias, the application of longitudinal sample weighting, and benchmarking key outcomes against representative counterparts. Additionally, we aimed to derive and discuss best practices for managing longitudinal studies, highlighting both strengths and challenges in conducting long-term follow-up studies on public mental health. An overarching aim was to offer valuable insights for refining research methods to improve participant retention and data quality in future longitudinal mental health and related studies.

## Methods and design

### Study design

In line with its research aims, the JEPSY study aimed to re-activate former KiGGS participants during the crucial transition to adulthood to encourage their re-participation. JEPSY therefore focused on individuals who took part in either the cross-sectional or longitudinal components of KiGGS Wave 2 and were 16 to 25 years old at the beginning of the JEPSY data collection period. For the implementation of the JEPSY study, all eligible individuals in the target group were re-contacted and invited to register in the RKI panel infrastructure Health in Germany and then take part in the subsequent JEPSY study. We calculated that a total of 2,200 JEPSY participants would be required to achieve sufficient power for the planned analyses. The integration of previously collected KiGGS data with newly collected JEPSY data establishes a strong foundation for future longitudinal analyses of mental health development in emerging adulthood. While such analyses are planned, they are beyond the scope of this paper and will be presented elsewhere. The database consisted of the second wave of the KiGGS study and the JEPSY study and was therefore well-suited to examine developmental trajectories from adolescence to adulthood and multidimensional factors that influence mental health.

### Participants/Study population

The KiGGS Wave 2 data came from 10,853 participants from the cohort and 14,464 from the cross-section of Wave 2 (559 cohort participants of Wave 2 were also newly drawn for the cross-section, so that for the purpose of analyses their data were added to the cross-sectional data as well). All KiGGS Wave 2 participants, including both cross-sectional and cohort participants, were considered for the JEPSY study if they met the following criteria: (i) they were aged 16 to 25 years at the beginning of the registration and data collection period on 1 March 2024; (ii) they (or their parents) had consented to their contact information being stored for potential re-contact; and (iii) no other factors were known that would preclude an invitation, such as being deceased, having permanently moved abroad, or having revoked consent.

### Recruitment and data collection process

The recruitment process consisted of two parts: (a) an invitation to register on the Health in Germany platform and (b) an invitation to take part in the JEPSY study.

- Registration process

Participant recruitment for registration comprised a postal invitation letter and two postal reminders sent 2 weeks apart to all those whose status was still open.

Invitees were asked to register on the Health in Germany online platform by creating a user account. They could access the registration form directly by scanning a personalized quick response (QR) code in the invitation letter with their smartphone or tablet or by entering a URL and access ID on a desktop computer.

By registering, users agreed that their contact details may be stored for the purpose of inviting them to JEPSY or other subsequent studies, with the option to revoke consent or delete their account themselves. For all invited persons, the contact details stored outside the user account were deleted after the recruitment of participants for registration had been completed.

b) Implementation of the JEPSY study

Invitations to the JEPSY study were sent out weekly via email to all new registrants. Participants who had not yet completed the online questionnaire received up to two reminder emails^2^, two weeks apart. The emails contained an individual link to the online questionnaire. Alternatively, it was possible to access the questionnaire from the user account after logging in. At the beginning of the questionnaire, respondents were asked for their consent to the JEPSY study. The questionnaire could only be completed online; paper questionnaires were not available. The registration form and the questionnaire were closed at the end of June 2024.

#### Strategies for enhancing participation

Various measures were implemented to improve participation. To ensure accurate re-contact, the home addresses of former participants from KiGGS Wave 2 were verified first, using an online interface provided by the Berlin population registry, which cross-references address data with local population registers across Germany. If a letter was undeliverable and returned to the RKI by the postal service, the address was verified again via the online interface and the letter resent, with or without a new address.

The invitation included a brochure containing the necessary information about Health in Germany, the JEPSY study, and compliance with data protection regulations. The materials (brochure and letters) were designed to be accessible, despite the complex nature of the content (e.g., two-stage recruitment, JEPSY as part of the DZPG). This was achieved by using clear, target group-oriented language, illustrative graphics, and a well-structured layout. Key information was visually highlighted.

Invitees were encouraged to learn more about Health in Germany and the JEPSY study on the project website and the institute’s website. They could also contact the RKI by email or via a toll-free telephone number to clarify any questions or decline participation.

The online registration and survey interfaces were designed to be responsive, so that they could also be easily completed on mobile devices. As a conditional incentive, participants were offered points worth 10 euros for registering and points worth 5 euros for completing the JEPSY questionnaire. The higher incentive for registration compared to questionnaire completion was intentional, as long-term engagement with the panel was prioritized over one-time participation in a single JEPSY survey wave. These points were credited to the participants’ user accounts and could be redeemed for various shopping vouchers. In the second email reminder for JEPSY, a note was added that, in consideration of the smaller number of cases, increased participation by men would be appreciated.

#### Quality assurance and training

Quality assurance played a key role in regular coordination meetings throughout the study implementation. Study processes, including participant recruitment for registration, were standardized based on standard operating procedures (SOPs). The SOPs were designed to ensure a professional and consistent telephone service for individuals invited to participate in the study. They defined clear procedures for data storage, entry and preparation, and standardized the process for enveloping and mailing invitation letters. The SOPs also ensured the rights of participants by outlining how personal data would be handled in accordance with data protection regulations, including procedures for proper data deletion or disclosure in response to information requests.

Staff members were trained according to these SOPs, with all training courses being monitored and evaluated by the quality assurance department to ensure adherence to the established quality management standards.

The questionnaire for participants was subjected to a pre-test. The quality of the data collected was ensured by filter guidance of the questionnaire and by defining ranges and soft prompts. Data cleaning and quality assurance involved ensuring the correct implementation of filtering procedures, identifying and correcting implausible information (e.g., out-of-range values, inconsistencies), and generating new variables as part of the data preparation process.

### Re-activation analysis

Longitudinal or panel studies face the problem of attrition (i.e., participants do not take part continuously across the survey waves or drop out permanently), so that the sample size decreases over time [33, 34]. The re-establishment of contact with former KiGGS participants was a different situation in that (a) two independent samples were re-contacted, (b) only one specific age group was recruited in each sample, (c) contact was established for a study that had not yet been planned at that time, (d) there was a change of the person giving consent (from the parents to the young adults), and (e) a relatively long period of time had passed since the last study. For this reason, the term ‘re-activation’ was preferred for the following, particularly because it draws attention to the prospect of re-establishing contact.

Re-activation analysis was conducted to identify factors that influenced both the likelihood of registration for the panel infrastructure and subsequent participation in the JEPSY study among individuals from KiGGS Wave 2. The aim of this analysis was to assess potential selection biases that may affect the generalizability of the study’s findings and interpretation of results.

For the analysis, variables were selected from the KiGGS Wave 2 data that could have had an impact on re-activation (i.e., the selection of variables was based on conceptual considerations). Two logistic regression models were conducted to predict two key outcomes: (1) registration for the panel Health in Germany, and (2) participation in JEPSY, indicating whether those who registered also participated in the study. Two separate models were constructed based on age groups, as some variables were only available for specific cohorts: one for participants under 11 years old and another for participants aged 11 and older at KiGGS Wave 2.

For participants aged 11 years and older, cohort membership was included to distinguish individuals who had participated already in the KiGGS baseline from those newly recruited in Wave 2. This distinction was important because cohort members, having been engaged with the study for a longer period, may have had a different likelihood of registering and participating in JEPSY compared to non-cohort members. Additionally, since cohort members had already participated in the KiGGS baseline study and were generally older (i.e., no 16/17-year-olds were represented), an interaction term between cohort membership and age was included to assess the combined effect of these two factors on study participation.

The following predictors were included in our logistic regression models:

- General health status: Derived from the Minimum European Health Module [35], self-reported or parent-reported, categorized into three groups: “excellent/very good” as the reference group, “good”, and “moderate/poor/very poor”.
- Mental health problem score: Summarized total score derived from the four subscales of emotional symptoms, conduct problems, hyperactivity/inattention, peer relationship problems, of the Strengths and Difficulties Questionnaire (SDQ) [36, 37], with parent-reported scores for children under 11 years old and self-reported scores for those from 11 years.
- Age: Measured in completed years.
- Sex assigned at birth: Binary variable with “male” as the reference group.
- SES: Three separate subscores derived from the KiGGS Economic Status Questionnaire [38], namely occupational status, educational status, income. These subscores were included separately as predictors in the logistic regression models. Lower values reflect a lower socioeconomic status (SES).
- Parents’ country of birth: Categorized as “both parents born in Germany”, “one parent born abroad”, or “both parents born abroad”, with “both parents born in Germany” as the reference category.
- Self-efficacy: Measured with the 10-item Self-Efficacy Scale [39].
- Social support: Measured with the three-item Social Support Scale [40].
- Cohort membership: Distinguishes individuals who participated in the KiGGS baseline survey from those recruited in Wave 2 (cross-section), with cross-sectional participation as the reference group.
- Interaction between age and cohort membership.

Subject to availability, for participants under 11 years old, the model included general health, the mental health problem score, age, sex assigned at birth, parents’ country of birth, and the SES subscores. For participants aged 11 and older, additional variables related to self-efficacy and social support were included, along with cohort membership and the interaction between age and cohort membership. The additional variables included in the model for participants from the age of 11 years was based on the research focus on mental health and the objective of being able to describe re-participation in emerging adulthood while considering central mental health outcomes and resources during adolescence.

### Calculation of the weighting variables

In cohort studies, one problem that can arise is due to selective (re-)participation: The original sample composition from a baseline survey becomes biased in follow-up surveys [41]. This can lead to distorted results. The aim of ‘sample weighting’ is to reduce selective participation effects, using information from KiGGS Wave 2. To balance out potential participation effects, the concept of inverse probability weighting was applied [42] to restore the (weighted) baseline sample at the time of KiGGS Wave 2. The respective weighting factor was derived from the inverse probability of re-participation in the follow-up survey. This probability was estimated, using known variables from the KiGGS Wave 2 survey through logistic regression (re-participation model). Thus, the inverse probability or longitudinal weighting factor 𝑤𝐿𝑆 for a participant 𝑖 was calculated as: $\:wL{S}_{i}=\frac{1}{{{\upvartheta\:}}_{\text{i}}}*wQ{S}_{i}$, where ϑ represents the predicted willingness to participate from the re-participation model, and 𝑤𝑄𝑆 is the cross-sectional weighting factor from KiGGS Wave 2, which adjusted the KiGGS Wave 2 sample to the population structure of 2014, based on factors such as age (in years), gender, federal state, German citizenship, and former parental education. The re-participation model was calculated separately for former cross-sectional participants and cohort participants, using the respective weighting factor. Additionally, the registration step and subsequent participation in JEPSY were modelled. For registration, the respective cross-sectional weight was used, and for JEPSY participation, the newly calculated longitudinal weight of registered individuals was applied in estimating the re-participation models.

The variable selection for estimating the individual re-participation models was implemented semi-automatically. Using a stepwise regression, variables from KiGGS Wave 2 were identified that were optimal for the model. For all variables, interactions by age, gender, and education of the mother were added. These variables and interactions were then checked for plausibility and actual significant effects. Variable levels were examined for cell size and extreme estimators/standard errors. Variables or interactions not plausible or significant were deleted, and the stepwise regression was restarted without the deleted variables. This process was repeated until an optimal model was identified. Only variables with a maximum missing proportion of 5% were considered. Missing values in continuous variables were automatically replaced by the median, and in categorical variables, by the mode. User defined missing values (not collected/filtered missing) were not automatically replaced but were, if appropriate, assigned to a category that corresponded most closely to the participation rate.

Since re-participation probabilities were estimated separately for the former cross-sectional and cohort participants, the separate samples needed to be combined. This was done using a convex combination, where the proportions of each sample were calculated based on the effective case numbers (rather than the nominal sample size) and merged accordingly. This approach had the advantage of increasing the effectiveness of the sample.

### Benchmarking of life satisfaction between JEPSY participants and representative counterparts

To characterize the JEPSY sample not only in relation to the initial KiGGS sample (for re-activation and weighting purposes) but also in comparison to a current representative sample in the same age group, data from another RKI study were used for benchmarking. Specifically, from January to May 2024, 62,623 adults (aged 16 years and older) in total, among them 7,761 emerging adults aged 16 to 25 years, participated in an RKI panel recruitment survey in order to register for a probabilistic panel sample (Health in Germany Panel) [14]. As part of a larger assessment protocol, participants answered the German version of a single-item satisfaction with life scale with an 11-point rating scale from not at all satisfied *(0)* to completely satisfied (10) [43], analogous to the JEPSY participants. For both samples, the mean value and 95% confidence interval were calculated. Sample weights were used to adjust the answers of participants of the recruitment survey to the current German population structure, and JEPSY participants were standardized according to their age, sex assigned at birth, educational level, and place of residence (West or East Germany), as an approximation of the recruitment survey sample composition.

## Results

### Registration process

**Fig. 1:**
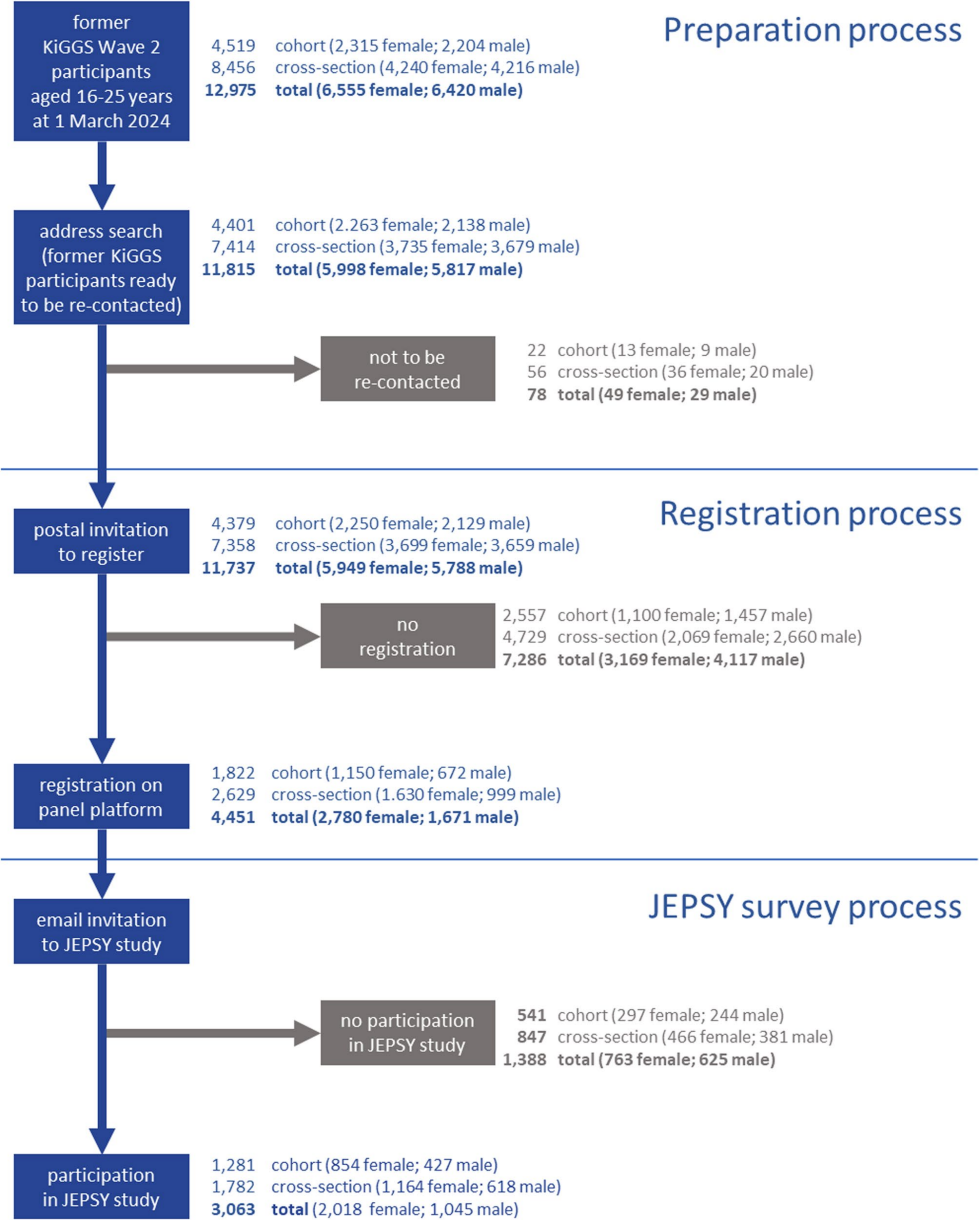
Number of Participants in the Re-Activation Process

Among the former participants in KiGGS Wave 2, a total of 12,975 individuals (6,555 female; 6,420 male) were identified (Fig. 1), all of whom were aged 16 to 25 years at the start of data collection (with all cohort members being at least 18 years old at the time). Of these, 4,519 were from the KiGGS cohort, and 8,456 were from the cross-sectional sample of Wave 2. Of the 12,975 participants, 11,815 (91.1%) had provided consent for re-contact (cohort: 4,401, 97.4%; cross-section: 7,414, 87.7%), with no known exclusions such as being deceased, having permanently moved abroad, or having withdrawn consent.

As a result of the prior home address verification, a total of 78 cases had to be excluded from invitation (Table 1) because eight of the individuals were deceased before 1 March 2024, 37 had permanently moved abroad, 16 had already been drawn for the probabilistic sample as part of Health in Germany [14], and 17 had a “restricted access” notice. The latter reason for exclusion meant that the population register may only provide information about the residential address if the person concerned is heard from beforehand.

Postal invitations to register were sent on 29 February 2024 to 11,737 people (cohort: 4,379; cross-section: 7,358). A total of 4,451 invitees (2,780 female; 1,671 male) registered on the platform until 30 June 2024 (cohort: 1,822; cross-section: 2,629).

A total of 7,286 people did not register (cohort: 2,557; cross-section: 4,729). Of these, only a few (60 cases) actively declined to register – in most cases, participation was refused without stating a reason (*n* = 22) – followed by the refusal to accept the letter (*n* = 12). The largest group of non-respondents (6,479 cases) were individuals from whom no feedback was obtained (all three letters were sent without any response from the invitees or the postal service). In 696 cases, there were problems with the delivery of the letters (e.g., one or several letters could not be delivered, so the letters were sent again with a new or unchanged address). A restricted access notice was received for 32 cases from a further address search. Finally, in 19 cases, the letters were returned because the invitees had permanently moved abroad (*n* = 15) or were deceased (*n* = 4).

Table 1 shows how these cases were classified as final disposition codes according to the standards of the American Association for Public Opinion Research [44]. On this basis, a recruitment rate of 37.9% was calculated (cohort: 41.7%; cross-section: 35.7%). There were differences between the sexes (i.e., the proportion of women registered was higher than for men: 46.7% vs. 28.9%).

**Table 1:** Final disposition codes (Preparation and Registration)

	**Preparation process**	**Registration process**	**total**
	***N***	***N***	***N***
(1) Registration at panel platform			
- Registered	--	4,451	4,451
(2) Eligible cases that were not registered (non-respondents)			
- Refusal	--	60	60
- Restricted access notice	17	32	49
(3) Cases of unknown eligibility			
- Nothing ever returned	--	6,479	6,479
- Unclear address status	--	696	696
(4) Not eligible			
- Deceased prior to 1 March 2024	8	4	12
- Permanently moved abroad	37	15	52
- Also drawn for probabilistic sample of Health in Germany	16	--	16
Total	78	11,737	11,815

### Implementation of the JEPSY survey

A total of 3,063 participants took part in the JEPSY survey. The completion rate (i.e., the proportion of registered participants who completed a full interview after receiving the email invitation) for the survey was 68.8%. There were substantial differences between the sexes in the completion rate: 62.6% of the male registered participants took part in the first survey. The completion rate of the female registered participants was 72.6%. Differentiated by age, there were less pronounced differences. For children who were between 6 and 9 years old at the time of the last KiGGS study, the completion rate was 65.2%. In the 10–14 years age group, it was 69.2%, and in the 15–19 age group, it was 69.9%. There were no major differences in the completion rate between sample types (cohort participants: 70.6%; cross-sectional participants: 67.8%).

### Re-activation analysis

The logistic regression models revealed several factors that influenced both registration for the panel and participation in JEPSY across the two age groups. Below, the findings for participants under 11 years of age (Table 2) and for those aged 11 and older (Table 3) are separately presented.

#### Odds of participation under 11 years of age

In the younger sample (children under 11 years), general health status and age did not significantly predict registration or participation in JEPSY (*p* > 0.05). However, the mental health problem score was a significant predictor. Higher mental health problems were negatively associated with both registration for the panel (OR = 0.95, 95% CI: 0.93–0.97) and participation in the JEPSY study (OR = 0.94, 95% CI: 0.92–0.96). This suggests that children with more pronounced mental health problems were systematically less likely to engage in re-participation in general.

Sex assigned at birth was a significant predictor of both outcomes. Female participants were significantly more likely to register for the panel (OR = 1.50, 95% CI: 1.25–1.80) and participate in JEPSY (OR = 1.65, 95% CI: 1.36–2.01) compared to males.

Higher occupational status was positively associated with both registration (OR = 1.13, 95% CI: 1.03–1.23) and participation (OR = 1.16, 95% CI: 1.05–1.28**)**, suggesting that children from families with higher occupational status were more likely to engage in JEPSY. The SES subscores for income and educational status were not significantly associated with either outcome (*p* > 0.05). Whether one or both parents were born abroad did not significantly affect registration or participation in this age group (*p* > 0.05).

#### Odds of participation aged 11 years and older

For participants aged 11 years and older, additional psychosocial factors were available and thus included in the model. Social support was a significant predictor of both registration and participation. Higher levels of social support were negatively associated with the likelihood of registering for the panel (OR = 0.99, 95% CI: 0.99–1.00) and participating in the JEPSY study after registration (OR = 0.99, 95% CI: 0.99–1.00), suggesting that individuals who perceived high levels of social support were less likely to engage with the study. The general health status was not a significant predictor for either registration or participation (*p* > 0.05). The mental health problem score was a significant negative predictor of both registration (OR = 0.98, 95% CI: 0.97–0.99) and participation OR = 0.98, 95% CI: 0.97–0.99), with higher scores corresponding to a lower likelihood of engaging in registration or participation in the JEPSY study.

Age was negatively associated with registration (OR = 0.97, 95% CI: 0.93–1.00),) but not with participation. Like the younger sample, among those 11 years of age and older, sex was a significant predictor, with females being significantly more likely to register (OR = 2.61, 95% CI: 2.37–2.88) and participate (OR = 2.85, 95% CI: 2.56–3.18) compared to males. Higher occupational status was associated with increased registration (OR = 1.08, 95% CI: 1.04–1.13) and participation (OR = 1.06, 95% CI: 1.01–1.11). Educational status was also positively associated with both registration (OR = 1.13, 95% CI: 1.08–1.17) and participation (OR = 1.13, 95% CI: 1.08–1.17). Income was not a significant predictor of either registration or participation. Cohort membership interacted significantly with age in this group (OR = 1.05, 95% CI: 1.00–1.10), indicating that older participants who were part of the original KiGGS cohort (KiGGS baseline) were more likely to register for the panel infrastructure, though this interaction was nonsignificant for participation. Participants with one parent born abroad were less likely to register and participate (registration: OR = 0.69, 95% CI: 0.58–0.83; participation: OR = 0.69, 95% CI: 0.57–0.84), as were those with both parents born abroad (registration: OR = 0.73, 95% CI: 0.61–0.88; participation: OR = 0.75, 95% CI: 0.61–0.92). It should also be noted that the presented models included fewer cases than the total of 11,815 invited individuals due to missing values in the included variables.

**Table 2:** Logistic regression model predicting panel registration and study participation for people under 11 years old

	**Registration**			**Participation**		
	OR^1^	95% CI^2^	*P* ^3^	OR^1^	95% CI^2^	*P* ^3^
General health status (ref. excellent/very good)						
Good	0.98	0.80-1.19	0.851	1.02	0.82-1.27	0.823
Moderate/poor/very poor	1.05	0.59-1.87	0.855	0.99	0.51-1.90	0.973
Mental health problem score	0.95	0.93-0.97	<.001	0.94	0.92-0.96	<.001
Age	1.02	0.94-1.12	0.616	1.08	0.98-1.19	0.119
Sex assigned at birth (ref. male)	1.50	1.25-1.80	<.001	1.65	1.36-2.01	<.001
SES^4^: Occupational status	1.13	1.03-1.23	0.008	1.16	1.05-1.28	0.003
SES^4^: Educational status	1.03	0.96-1.11	0.431	1.01	0.93-1.10	0.749
SES^4^: Income	1.04	0.98-1.11	0.194	1.05	0.98-1.12	0.115
Parents' country of birth (ref. both parents born in Germany)						
One parent born abroad	0.94	0.69-1.29	0.718	0.98	0.70-1.38	0.918
Both parents born abroad	0.95	0.67-1.36	0.796	1.27	0.87-1.85	0.214
Observations		2,127			2,127	

^1^*OR* Odds ratio

^2^*CI* Confidence interval

^3^*p* p-value

^4^*SES* Socioeconomic status

**Table 3:** Logistic regression models for predicting panel registration and study participation for people 11+ years old

	**Registration**			**Participation**		
	OR^1^	95% CI^2^	*P* ^3^	OR^1^	95% CI^2^	*P* ^3^
General health status (ref. excellent/very good)						
Good	0.97	0.87-1.07	0.512	0.93	0.83-1.04	0.218
Moderate/poor/very poor	0.86	0.73-1.03	0.098	0.83	0.69-1.00	0.056
Mental health problem score	0.98	0.97-0.99	0.003	0.98	0.97-0.99	0.004
Social support	0.99	0.99-1.00	<.001	0.99	0.99-1.00	<.001
Self-efficacy	1.00	1.00-1.01	0.124	1.00	1.00-1.01	0.871
Age	0.97	0.93-1.00	0.046	0.97	0.93-1.01	0.095
Sex assigned at birth (ref. male)	2.61	2.37-2.88	<.001	2.85	2.56-3.18	<.001
SES^4^: Occupational status	1.08	1.04-1.13	<.001	1.06	1.01-1.11	0.014
SES^4^: Educational status	1.13	1.08-1.17	<.001	1.13	1.08-1.17	<.001
SES^4^: Income	0.99	0.96-1.03	0.740	1.02	0.99-1.06	0.191
Parents' country of birth (ref. both parents born in Germany)						
One parent born abroad	0.69	0.58-0.83	<0.001	0.69	0.57-0.84	<.001
Both parents born abroad	0.73	0.61-0.88	0.001	0.75	0.61-0.92	0.005
Cohort membership (ref. cross-sectional participant)	0.68	0.35-1.34	0.265	0.84	0.41-1.75	0.649
Cohort membership X Age	1.05	1.00-1.10	0.042	1.03	0.98-1.09	0.209
Observations		8,093			8,093	

^1^*OR* Odds ratio

^2^*CI* Confidence interval

^3^*p *p-value

^4^*SES* Socioeconomic status

### Weighting

The logistic model estimations for panel registration and JEPSY participation, including the final variable list for both the cross-sectional and cohort samples, are presented in the Additional file 1. In addition to sociodemographic and health parameters, technical variables (e.g., participation mode and questionnaire type) were identified with the stepwise selection method. These variables were subsequently used to estimate the probability of re-participation.

To evaluate the weighting, selected reference distributions from the KiGGS2 survey were compared with the distributions from the JEPSY sample. Additionally, the effectiveness and dispersion of the weights were calculated to assess the impact of the adjustments on the statistical reliability of the sample.

Table 4 presents the unweighted and weighted proportions of selected parameters within the KiGGS Wave 2 sample. The reference group comprised all KiGGS Wave 2 participants included in the JEPSY sampling frame, compared to those who participated in the JEPSY study. Effective weighting should minimize discrepancies between the weighted proportions. Concerning the sociodemographic characteristics, the unweighted proportions revealed notable differences, particularly in sex assigned at birth, parents’ country of birth, and educational status. In contrast, when examining the weighted distributions, these differences were effectively reduced to an acceptable level. However, for the former KiGGS Wave 2 age groups, the discrepancies between the weighted samples increased. The two health-related parameters (SDQ Total Difficulties Score and General Health Status) showed no substantial differences in either the unweighted or the weighted distributions.

**Table 4:** Comparison of selected KiGGS Wave 2 outcomes between the KiGGS and JEPSY samples

**KiGGS Wave 2 Parameter**		**KiGGS Wave 2 Unweighted [%] (CI)** ^**a**^	**JEPSY Unweighted [%] (CI)** ^**a**^	**KiGGS Wave 2 Weighted [%] (CI)** ^**a**^	**JEPSY Weighted [%] (CI)** ^**a**^
Sex assigned at birth	Male	49.5 (48.6-50.3)	34.1 (32.5-35.8)	52.1 (51.0-53.3)	49.7 (46.7-52.6)
	Female	50.5 (49.7-51.4)	65.9 (64.2-67.5)	47.9 (46.7-49.0)	50.3 (47.4-53.3)
Age group	6-9 years	15.0 (14.4-15.6)	13.7 (12.6-15.0)	15.6 (14.8-16.5)	9.1 (7.6-10.8)
	10-13 years	42.7 (41.8-43.5)	43.4 (41.7-45.2)	41.1 (40.0-42.2)	40.0 (37.2-42.9)
	14-17 years	41.0 (40.2-41.9)	41.2 (39.5-42.9)	42.1 (41.0-43.3)	47.0 (44.0-49.9)
	18-31 years	1.3 (1.1-1.5)	1.7 (1.3-2.2)	1.2 (0.9-1.4)	3.9 (2.7-5.6)
CASMIN^b^ educational groups (maximum of parents in the household)	Basic education	10.2 (9.7-10.8)	5.9 (5.1-6.8)	23.4 (22.2-24.6)	20.7 (17.7-24.1)
	Intermediate education	54.2 (53.3-55.1)	50.7 (49.0-52.5)	50.1 (48.9-51.2)	52.2 (49.1-55.2)
	Higher education	35.6 (34.7-36.4)	43.4 (41.7-45.1)	26.6 (25.7-27.5)	27.1 (24.9-29.4)
Parents’ country of birth	Both parents born in Germany	82.7 (82.0-83.3)	86.7 (85.5-87.9)	74.2 (73.1-75.4)	77.4 (74.4-80.2)
	One parent born abroad	8.2 (7.7-8.7)	6.7 (5.9-7.7)	10.1 (9.4-10.9)	8.5 (6.8-10.5)
	Both parents born abroad	9.1 (8.6-9.6)	6.6 (5.7-7.5)	15.7 (14.7-16.7)	14.1 (11.7-16.9)
General health status (categorical)	Very good/good	95.9 (95.6-96.3)	96.5 (95.8-97.1)	95.5 (95.0-96.0)	95.6 (94.1-96.7)
	Average/poor/very poor	4.1 (3.7-4.4)	3.5 (2.9-4.2)	4.5 (4.0-5.0)	4.4 (3.3-5.9)
SDQ total difficulties score, international norms	Unremarkable	87.5 (86.8-88.1)	88.2 (86.8-89.4)	87.2 (86.3-88.1)	86.1 (83.5-88.4)
	Borderline	8.8 (8.3-9.4)	8.5 (7.4-9.6)	8.9 (8.1-9.7)	9.4 (7.6-11.6)
	Conspicuous	3.7 (3.3-4.1)	3.4 (2.7-4.2)	3.9 (3.4-4.5)	4.5 (3.2-6.3)

^a^*CI* Confidence interval

^b^*CASMIN* Classification (Comparative Analyses of Social Mobility in Industrial Nations) [45]

The metrics presented in Table 5 enabled the assessment and comparison of the dispersion of the weights. Dispersion is indicated by the minimum, median, maximum, and the coefficient of variation (CV) of the weights. A higher dispersion typically increases the statistical uncertainty of estimates. To evaluate and compare the effectiveness of the weighting, the Kish formula for calculating effective sample size was used [46]. Effectiveness was defined as the ratio of the effective sample size, considering the weights, to the sample size without weighting. For example, an effectiveness of 50% indicated that the effective sample size was half of the actual sample size. For the KiGGS Wave 2 sample, the effectiveness, which accounts for cross-sectional and for the cohort sample also for longitudinal weighting, was 57.31%. When longitudinal weights for registration and JEPSY participation were applied, the effectiveness dropped to 33.80%. The higher dispersion in the JEPSY sample was reflected in the larger differences between the minimum and maximum values, as well as the increased CV.

**Table 5:** Indicators of weighting

	**Minimum**	**Median**	**Maximum**	**CV**	**Effectiveness**
Unweighted	1	1	1	0	100.000
KiGGS Wave 2 weight	0.06461	0.76016	8.39212	86.316	57.31
JEPSY weight	0.03246	0.54442	9.64756	139.961	33.80

*CV* Coefficient of variation

### Benchmarking of life satisfaction between JEPSY participants and representative counterparts

The comparison of the mean general life satisfaction scores between the re-activated JEPSY sample and the Health in Germany panel data showed no statistically significant differences (with weighted survey data). The mean life satisfaction score in the re-activated JEPSY sample was 6.61 (std. err. = 0.084, 95% CI [6.44, 6.77]), while the mean score in the Health in Germany panel was 6.75 (std. err. = 0.034, 95% CI [6.68, 6.81]). Moreover, the overlapping confidence intervals indicated a comparable distribution of responses for this measure of well-being. A t-test for independent samples was also conducted to ascertain whether there was a statistically significant difference between the samples. The results demonstrated no statistically significant difference between the samples, t(11,661) = 1.51, *p* = 0.131.

## Discussion

This study focused on the methodological process and success of integrating former child and adolescent study participants into a newly established online panel infrastructure, providing key insights into the feasibility and challenges of re-activation.

### Registration and participation rates

One important aim of the re-activation was to attract as many former participants of KiGGS Wave 2 (in the relevant age group) as possible to register, knowing that not all those registered would participate in JEPSY and that a high number of participants was beneficial for the analyses. The targeted minimum number of approximately 2,200 was significantly exceeded with 4,451 registered. However, the number of participants in the JEPSY survey was considerably lower, at 3,063. This is presumably because registration was non-binding (i.e., the incentive for registration was also granted if it was not followed by JEPSY participation). This decision was made because– independent of JEPSY– as few participants as possible in the KiGGS cohort were to be lost with regard to a potential continuation of the entire KiGGS cohort study.

The overall recruitment rate (37.9%) suggests there was still an interest in the study among the former participants– even though the study was discontinued many years back. This result could be due to a lasting ‘study bond’. Another reason could be that the panel infrastructure enables an uncomplicated study engagement or that a conditional incentive was granted for registration regardless of whether they took part in the survey. The fact that only a few people actively refused and a large proportion of those invited did not reply indicates that perhaps more people could have been attracted to register and participate. In previous RKI studies, telephone calls were implemented to reach undecided participants, which could have increased participation rates here as well, but such measures were not feasible this time due to monetary and personnel restrictions. Unfortunately, in most cases, the reasons for non-participation are unknown. Perhaps the target people had other priorities during life transitions with this age group or that the letters simply did not reach the individuals directly, but their parents or former co-residents instead. Additionally, the extended gap of up to 9 years since the last study participation likely weakened the sense of connection to the study, rendering the contact process more akin to an initial recruitment. However, these findings highlight the need for developing targeted engagement strategies, particularly for harder-to-reach subgroups. Potential approaches could include multimodal contact strategies (e.g., combining postal invitations with SMS or email reminders, where feasible), feedback mechanisms, such as providing summarized study findings or offering personal feedback. Additionally, testing alternative incentive models tailored to the preferences of different subgroups could also be a valuable approach.

### Re-activation analysis

Our re-activation analysis revealed several key factors that influenced both registration for and participation in JEPSY. Mental health problems during childhood and adolescence, as measured by the SDQ collected during childhood or adolescence, were a significant predictor of a lower likelihood of re-activation in young adulthood. Participants with higher SDQ problem scores, reflecting early emotional and behavioural difficulties, were less likely to register for the panel and participate in the JEPSY study. This highlights the role of early-life mental health in shaping long-term engagement with research studies.

Interestingly, these findings appear to diverge from studies in the Netherlands, which reported minimal associations between diagnosed mental disorders and re-participation rates [47–49]. However, regarding depressive symptoms, higher symptom severity correlated with lower participation rates over time– a trend similarly seen with anxiety symptoms [50]. This alignment suggests that the SDQ, while not capturing formal diagnoses, may serve as an early indicator of subclinical emotional and behavioural problems, like depressive or anxiety symptoms, which might be more predictive of re-activation difficulties in young adulthood. One possible explanation is that participants with elevated SDQ scores may experience more barriers to participation, such as reduced motivation, difficulties with organization, or avoidance of activities that may increase their emotional burden. These factors can make it more challenging to engage this group in follow-up research, potentially leading to selective re-activation rates and a biased sample that underrepresents those with poorer mental health at baseline.

Given these findings, targeted strategies to reduce barriers for participants with mental health problems are essential. Empirical evidence from clinical research highlights that individuals with serious mental illness (SMI) often face a range of challenges that reduce their likelihood of sustained study participation. These include logistical difficulties such as lack of transportation, limited phone access, competing appointments, or ongoing mental and physical health issues [51]. Drawing on these insights, strategies such as flexible scheduling, proactive contact management (e.g., using up-to-date health records), and mental health-sensitive communication approaches may help reduce dropout among psychologically vulnerable groups. Addressing these barriers is critical to mitigating bias introduced by selective re-activation rates and ensuring that longitudinal mental health studies accurately represent individuals with varying levels of mental well-being at baseline.

Sex differences were consistently observed across both age groups, with females being more likely to register and participate than males. This pattern aligns with prior research on survey participation that indicated that women generally show higher response rates [52, 53], even if there were a few indications of a higher willingness among young men in health research and especially in online studies [54, 55]. Suitable measures to increase young men’s willingness to participate are yet to be identified. Understanding these sex differences is important for tailoring recruitment strategies and ensuring balanced representation of both sexes in longitudinal studies.

SES also played a significant role, particularly occupational status and education, in predicting registration and participation. Participants from families with higher occupational or educational statuses were more likely to register and complete the survey, a pattern consistent with findings on education bias in surveys (e.g [56]). This emphasizes the need to account for SES in the design and analysis of longitudinal studies, as it may influence participation and potentially bias results. Families with a low occupational status and/or educational level may require specific recruitment methods or incentives to increase participation. Implementing such strategies to enhance accessibility for lower SES groups could help mitigate this issue.

In addition to recruitment strategies, the design of the survey itself played a role in enhancing accessibility for participants from lower SES backgrounds. To reduce respondent burden, the questionnaire was deliberately kept brief (approximately 15 min), and shorter validated instruments such as the PHQ-2 were used instead of longer versions. Visual analogue scales were employed for some constructs to reduce cognitive complexity. While no cultural adaptations or interviewer-administered formats were implemented, participants had access to a study hotline for clarification and support. This pragmatic approach sought to balance scientific rigor with feasibility and participant burden. Nonetheless, it is possible that limited health literacy among some participants still posed challenges, particularly when interpreting more abstract or psychological items. These considerations should inform the development of future surveys aiming to reach underrepresented or socioeconomically disadvantaged populations.

An unexpected finding was the association between higher levels of social support in childhood and lower registration and participation rates in young adulthood. Perhaps individuals who experienced strong social support during childhood may perceive less of a need for additional external help or intervention in adulthood, thus feeling less compelled to participate in studies related to mental health. Their established social networks might offer sufficient coping mechanisms, which could render study participation less personally relevant [57].

Moreover, high social support in childhood may have contributed to fewer adverse or traumatic life events, leading to better mental health outcomes. As a result, these individuals might face fewer psychological challenges in young adulthood, reducing the perceived necessity of participating in a study focused on mental health [58, 59]. From this perspective, lower participation among individuals with high early social support may reflect psychological resilience rather than vulnerability.

This pattern might appear counterintuitive when juxtaposed with the finding that individuals with elevated SDQ scores—indicative of early emotional and behavioural difficulties—also showed reduced participation. However, both effects may be explained by different underlying mechanisms: while early psychological difficulties may lead to motivational, cognitive, or emotional barriers to participation, high early social support might reduce the perceived relevance of mental health research. Rather than representing a contradiction, these findings highlight two distinct pathways—resilience and vulnerability—through which early-life experiences shape engagement in longitudinal mental health research.

Interestingly, our results highlight the role of cohort membership in influencing re-activation. While participants from the earlier KiGGS baseline cohort were generally not significantly more likely to register (OR = 0.68, *p* = 0.265), the interaction effect with age (OR = 1.05, *p* = 0.042) suggested that the negative effect of cohort membership on registration decreased with increasing age. This indicates that older cohort members were more likely to register compared to younger participants, possibly reflecting a stronger sense of connection or loyalty developed through repeated participation. Despite this, the registration effect did not translate proportionally into participation in the JEPSY survey, likely due to factors such as competing life demands or reduced motivation. The extended time gap since the last wave may have further exacerbated this, as the reactivation process resembled initial recruitment more closely than traditional follow-up.

Implementing longitudinal studies in the field of public mental health poses unique challenges, particularly in maintaining participant engagement across life transitions. Best practices for addressing these challenges include maintaining regular contact, providing flexible participation options, and offering meaningful incentives. These approaches can help enhance retention rates and improve data quality [3, 60, 61]. The findings of this study highlight the need for refined retention strategies, particularly for groups who are underrepresented, such as individuals with early psychosocial difficulties, lower SES, or limited social motivation. Financial and staffing limitations restricted the ability to implement additional follow-up contacts or more tailored recruitment approaches, which could have targeted these hard-to-reach groups more effectively. Moreover, the focus of this study was to assess the feasibility of re-engaging participants within the existing infrastructure, and the use of more personalized, resource-intensive strategies was not feasible within this scope. Future research should explore how these strategies can be integrated into larger studies, with adequate support and funding, to increase engagement and retention among these underrepresented groups. These strategies could include additional follow-up contacts, more tailored recruitment approaches, and incentives designed specifically to encourage participation among hard-to-reach groups.

### Weighting

One way to address biases caused by selective participation is the use of inverse probability weighting applied to an entire study sample. An advantage of this method is that the calculated weighting factors can be made available, along with the dataset. This enables researchers to account easily for potential bias, at least in sensitivity analyses, such as comparing weighted and unweighted results within specific projects. As shown, the primary purpose of the weighting factors was to adjust the JEPSY sample to match the original composition of the KiGGS Wave 2 study sample. This adjustment reduced potential biases due to selective participation, as indicated by very similar distribution rates of weighted JEPSY data with KiGGS Wave 2 (Table 4). However, researchers should be aware that this correction comes at the cost of increased variance. When using longitudinal weighting factors, statistical uncertainty increases accordingly. Another critical point is that the KiGGS Wave 2 parameters used for modelling re-participation dated back significantly and were partially based on parental reports. This raises questions about the suitability of these parameters for accurately predicting current willingness to participate. Nevertheless, by working with longitudinal data, researchers should always check the sample composition within their specific project, even with the use of weighting factors. Since the weighting is calculated for the entire sample, it is possible that the weighted distribution for specific topics may not align with an original distribution. In such cases, researchers might consider topic-specific approaches or project-related adjustments to account for non-response and ensure more accurate representation.

### Benchmarking

Our benchmarking analysis of general life satisfaction suggested that the re-activated JEPSY sample aligned well with the representative data from the Health in Germany panel, at least concerning the key indicator of subjective well-being. The similarity in mean life satisfaction scores between the JEPSY sample and the Health in Germany panel indicates that the recruitment and weighting processes were effective at mitigating selection biases, at least with respect to this specific measure. However, the lack of comparable mental health indicators limits broader generalizations regarding the representativeness of other mental health domains. Future studies could benefit from harmonizing indicator measurement across age groups and panels to enable a more comprehensive benchmarking of re-activated samples. Nonetheless, our findings support the validity of the JEPSY data for analyses involving subjective well-being and related outcomes.

### Strengths and limitations

One of the major study strengths is the scalability of the re-activation framework. The use of a structured, two-step recruitment process allowed for the efficient engagement of participants across a wide geographical area, demonstrating the viability of combining traditional postal invitations with digital platforms.

Another notable strength was the integration of pre-existing KiGGS Wave 2 data with new data collected through the “Health in Germany” panel. This methodological design leveraged former datasets to answer longitudinal research questions without requiring additional baseline data collection.

Furthermore, this study demonstrates the potential of linking former cohort data with modern digital infrastructures, which can streamline future longitudinal studies and reduce operational costs. Still, our methodology had several limitations.

One problem was that for many invitees who did not attend, no feedback was available, and it is not entirely clear whether they received the invitation letter, and, if so, why they did not participate. Future studies should consider using multiple contact methods and enhancing engagement through personalized communication. Also, selective re-activation emerged as a key challenge, with lower response rates among males, individuals with lower SES, and those with higher mental health problem scores. While weighting adjustments were applied to address these biases, the reliance on former KiGGS Wave 2 data for estimating participation probabilities represents a limitation. This historical data may not fully reflect the participants’ current circumstances, potentially limiting the effectiveness of the adjustments in correcting for all sources of bias. Another limitation was the potential for residual bias despite weighting adjustments [62].

Additionally, our benchmarking process, which compared the re-activated sample with a contemporary population-based panel, was constrained by the availability of comparable measures. Life satisfaction was the only indicator available for direct benchmarking, which limits the extent to which broader generalizations about the representativeness of the sample can be made. Future studies would benefit from harmonizing indicators across datasets to enable more comprehensive benchmarking and validation of findings. Finally, the push-to-online panel recruitment strategy, while effective for engaging young adults, also introduced potential limitations. Former KiGGS Wave 2 participants with limited access to or familiarity with online platforms may have been inadvertently excluded.

## Conclusions

The JEPSY study is a good example of the integration of existing samples into a modern online panel infrastructure with the aim of linking existing data with newly collected data and making it available in a timely manner for the investigation of population-based cross-sectional and longitudinal research questions. The additional potential for epidemiological public health research is that the online panel infrastructure considerably facilitates further longitudinal data collection and retention measures. However, reactivating existing samples via the postal route was initially costly and time-consuming, and, especially with special target groups such as young adults in this example, it was important to minimize or compensate for any selection effects, using suitable measures. Overall, the JEPSY study offers valuable insights into factors influencing participation in longitudinal public mental health research. By understanding the determinants of participation and implementing targeted retention strategies, future studies can improve the quality and generalizability of findings from public mental health research.

## Supplementary Information

Additional file 1: Logistic model estimations for registration and participation, including the final variable list for both the cross-sectional and cohort samples.

## Data Availability

A scientific use file of the KiGGS survey data is available upon request; the JEPSY survey data are subject to an embargo of 12 months from the end of data collection. Once the embargo expires, the data will be made available on request to the Secure Data Centre at the Robert Koch Institute for non-commercial research. Requests should be directed to fdz@rki.de.

## Abbreviations

DZPG: German Centre for National Mental Health [Deutsches Zentrum für psychische Gesundheit]
JEPSY: Study on Mental Health in Emerging Adulthood [Studie zur psychischen Gesundheit von jungen Erwachsenen in Deutschland]
KiGGS: German Health Interview and Examination Survey for Children and Adolescents [Studie zur Gesundheit von Kindern und Jugendlichen in Deutschland]
RKI: Robert Koch Institute

## Footnotes

1 The migration background variable was based on the child’s and parents’ birthplaces and nationalities. For single-parent households, the status of the custodial parent was applied. If data were missing, the custodial parent’s status, the child’s report, or the language spoken at home were used as proxies [6]. The recommendations for the collection and analysis of migration-related determinants have since been updated [7].

2 A third reminder was sent to people in the greater Berlin area to increase the number of individuals eligible for a follow-up project.
