# Supplementary material for: Integration of former child and adolescent study participants into a national health online panel for a longitudinal study on young adult mental health
Source: BMC Med Res Methodol. 2025 Jul 1;25:172. doi: 10.1186/s12874-025-02613-2 (PMC12211355; doi:10.1186/s12874-025-02613-2)
Supplement: Supplementary file 1 — Additional file 1: Logistic model estimations for registration and participation, including the final variable list for both the cross-sectional and cohort samples. [file 12874_2025_2613_MOESM1_ESM.docx]

Additional file 1

| **Registration** | | | | | | | | | | | | | | | |  |  |
| --- | --- | --- | --- | --- | --- | --- | --- | --- | --- | --- | --- | --- | --- | --- | --- | --- | --- |
| **Cross-Sectional** | | | | | **Cohort** | | | | | | | | | | |  |  |
| **Parameter** | | **Estimate (CI)^1^** | | **p-value** | **Parameter** | | | | | **Estimate (CI)^1^** | | | **p-value** | |  |  |  |
| Intercept |  | -0.8607  (-1.6512--0.0702) | 0.0328 | | Intercept |  | | | 1.0741  (-5.0203-7.1685) | | | 0.7297 | | |  |  |  |
| Gender | Female | REF |  | | Gender | female | | | REF | | |  | | |  |  |  |
|  | Male | -1.1046  (-1.6318--0.5774) | <0.0001 | |  | male | | | 0.3012  (-0.4907-1.093) | | | 0.4559 | | |  |  |  |
| KiGGS age group | 6-9 years | REF |  | | KiGGS age group | 18-31 years | | | REF | | |  | | |  |  |  |
|  | 10-13 years | -0.1004  (-0.2674-0.0665) | 0.2384 | |  | 10-13 years | | | -3.6707  (-10.3219-2.9804) | | | 0.2793 | | |  |  |  |
|  | 14-17 years | -0.2246  (-0.3985--0.0508) | 0.0113 | |  | 14-17 years | | | -3.7896  (-10.4326-2.8534) | | | 0.2634 | | |  |  |  |
| CASMIN - Educational groups (maximum of parents in the household) | Intermediate education | REF |  | | CASMIN - Educational groups (maximum of parents in the household) | Intermediate education | | | REF | | |  | | |  |  |  |
|  | Higher education | 0.1068  (-0.0329-0.2465) | 0.1341 | |  | Higher education | | | 0.2948  (0.1088-0.4809) | | | 0.0019 | | |  |  |  |
|  | Basic education | -0.3265  (-0.5581--0.0949) | 0.0057 | |  | Basic education | | | -0.2755  (-0.5519-0.001) | | | 0.0508 | | |  |  |  |
| Tetanus: Booster vaccination by the end of the 7th year of life | No booster vaccination until the end of the 7th year of life | REF |  | | Age of biological mother at birth (categorical) | Up to 24 years | | | REF | | |  | | |  |  |  |
|  | Booster vaccination until the end of the 7th year of life | -0.2837  (-0.5447--0.0227) | 0.0332 | |  | 35+ years | | | 0.3047  (-0.0138-0.6232) | | | 0.0607 | | |  |  |  |
| Tetanus vaccination status at the time of examination | Vaccinated, but not sufficiently | REF |  | |  | 30-34 years | | | 0.2603  (-0.0368-0.5574) | | | 0.0860 | | |  |  |  |
|  | Sufficiently vaccinated | 0.5754  (0.1492-1.0017) | 0.0082 | |  | 25-29 years | | | 0.1096  (-0.2033-0.4225) | | | 0.4924 | | |  |  |  |
| Ever had measles (self-reported) | No | REF |  | | Ever obesity (doctor's diagnosis) | No | | | REF | | |  | | |  |  |  |
|  | Yes | -0.7941  (-1.2556--0.3325) | 0.0007 | |  | Yes | | | -0.5648  (-1.0234-0.1061) | | | 0.0158 | | |  |  |  |
| Birch (pollen) (t3) sensitization | No (CAP class = 0) | REF |  | | Father's behavior: Teaches me things | Applies somewhat | | | REF | | |  | | |  |  |  |
|  | Yes (CAP class >=1) | 0.9451  (0.4611-1.429) | 0.0001 | |  | Applies little | | | 0.1904  (-0.0971-0.4779) | | | 0.1943 | | |  |  |  |
| Birch (pollen) (t3) sensitization X Gender | Yes (CAP class >=1) X male | -0.8249  (-1.4742--0.1756) | 0.0128 | |  | Applies completely | | | -0.233  (-0.4154-0.0505) | | | 0.0123 | | |  |  |  |
| Participation in the mental health module (Bella) | No | REF |  | |  | Does not apply | | | -0.9117  (-1.4224-0.4009) | | | 0.0005 | | |  |  |  |
|  | Yes | 0.8004  (0.3225-1.2784) | 0.001 | | Nutrition questionnaire | No | | | REF | | |  | | |  |  |  |
|  | undefined | 0.2839  (-0.1798-0.7477) | 0.2301 | |  | Yes | | | 1.3884  (0.807-1.9698) | | | <0.0001 | | |  |  |  |
| Foreign nationality (according to the residents' registration office) | Non-foreigner | REF |  | | Obedient/does what is asked | Partially applies | | | REF | | |  | | |  |  |  |
|  | Foreigner | -0.6331  (-1.0209--0.2453) | 0.0014 | |  | Does not apply | | | 0.503  (0.0775-0.9285) | | | 0.0205 | | |  |  |  |
| Participation in the motoric module (MoMo) | No | REF |  | |  | Definitely applies | | | 0.3307  (0.1478-0.5135) | | | 0.0004 | | |  |  |  |
|  | Yes | 0.7173  (0.4324-1.0022) | <0.0001 | | Parental residency status | Permanent | | | REF | | |  | | |  |  |  |
|  | undefined | -0.5427  (-0.8344--0.2509) | 0.0003 | |  | German or EU citizen | | | 0.3495  (-0.0514-0.7505) | | | 0.0875 | | |  |  |  |
| Participation in the MoMo module X Gender | undefined X male | 0.3979  (-0.0584-0.8542) | 0.0874 | |  | Temporary | | | -1.1197  (-2.4471-0.2076) | | | 0.0982 | | |  |  |  |
|  | Yes X male | 0.0594  (-0.3819-0.5006) | 0.7919 | | Participation in the motoric module (MoMo) | No | | | REF | | |  | | |  |  |  |
| Participation in the environmental module (GerES) | No | REF |  | |  | Yes | | | 0.5797  (0.256-0.9034) | | | 0.0005 | | |  |  |  |
|  | Yes | 0.305  (0.0503-0.5596) | 0.0189 | |  | undefined | | | 0.2747  (-0.042-0.5914) | | | 0.0891 | | |  |  |  |
| Age of biological mother at birth (categorical) | Up to 24 years | REF |  | | Presumed highest educational level of your child | Middle school diploma (MSA), secondary school certificate | | | REF | | |  | | |  |  |  |
|  | 35+ years | 0.3441  (0.0937-0.5946) | 0.0071 | |  | Abitur, general higher education entrance qualification | | | 0.3284  (0.1284-0.5284) | | | 0.0013 | | |  |  |  |
|  | 30-34 years | 0.1697  (-0.0623-0.4016) | 0.1516 | |  | Vocational Abitur, specialized higher education entrance qualification | | | 0.1691  (-0.1182-0.4564) | | | 0.2487 | | |  |  |  |
|  | 25-29 years | 0.1036  (-0.1371-0.3443) | 0.3988 | |  | Secondary school diploma | | | -1.1623  (-1.7078-0.6168) | | | <0.0001 | | |  |  |  |
| Smoking yes/no (mother) | No | REF |  | | Rubella: first vaccination by the end of the 24th month of life | No vaccination by the end of the 2nd year of life | | | REF | | |  | | |  |  |  |
|  | Yes | -0.1747  (-0.345--0.0044) | 0.0443 | |  | First vaccination by the end of the 2nd year of life | | | 0.42  (0.1643-0.6757) | | | 0.0013 | | |  |  |  |
| Monthly household net income (categorical) (imputed) [€] |  | 0.0391 (0.01-0.0682) | 0.0084 | | SDQ Peer problems, international standardization | Unconspicuous | | | REF | | |  | | |  |  |  |
| Participation type | Examination and Survey | REF |  | |  | Conspicuous | | | 0.4527  (0.1633-0.7422) | | | 0.0022 | | |  |  |  |
|  | Survey | -0.5015  (-0.9465--0.0566) | 0.0272 | |  | Borderline | | | 0.1814  (-0.1128-0.4755) | | | 0.2267 | | |  |  |  |
| SDQ externalizing problems - Cutoff | No | REF |  | | Züricher Kurzfragebogen zum Erziehungsverhalten (ZKE) subscale: Rules |  | | | -0.0521  (-0.0867-0.0176) | | | 0.0031 | | |  |  |  |
|  | Yes | -0.3927  (-0.6245--0.161) | 0.0009 | | Nutrition questionnaire X Gender | Yes | | | -1.1272  (-1.9367-0.3177) | | | 0.0064 | | |  |  |  |
| Often unhappy/depressed/cries frequently | Partially applies | REF |  | |  |  | | |  | | |  | | |  |  |  |
|  | Definitely applies | 0.5843 (0.0747-1.0939) | 0.0246 | |  |  | | |  | | |  | | |  |  |  |
|  | Does not apply | -0.1407  (-0.342-0.0606) | 0.1706 | |  |  | | |  | | |  | | |  |  |  |
| Easily distracted/inattentive | Partially applies | REF |  | |  |  | | |  | | |  | | |  |  |  |
|  | Does not apply | 0.298  (0.1595-0.4364) | <0.0001 | |  |  | | |  | | |  | | |  |  |  |
|  | Definitely applies | 0.0079  (-0.2364-0.2522) | 0.9493 | |  |  | | |  | | |  | | |  |  |  |
| Contact with farm or stable animals at ages 0-6 years | No | REF |  | |  |  | | |  | | |  | | |  |  |  |
|  | Yes | -0.2295  (-0.3851--0.0739) | 0.0039 | |  |  | | |  | | |  | | |  |  |  |
| **Participation** | | | | | | | | | | | | | | | |  |  |
| **Cross-Sectional** | | | | | **Cohort** | | | | | | | | | | |  |  |
| **Parameter** | | **Estimate (CI)^1^** | | **p-value** | **Parameter** | | | | | **Estimate (CI)^1^** | | | **p-value** | | | |  |
| Intercept |  | 7.8492  (-1.9908-17.6891) | | 0.1179 | Intercept | |  | | | 0.5611  (-0.3841-1.5063) | | | 0.2445 | | | |  |
| Gender | Female | REF | |  | Gender | | Female | | | REF | | |  | | | |  |
|  | Male | -0.3839  (-0.6462- -0.1216) | | 0.0041 |  | | Male | | | -0.7203  (-1.006-0.4345) | | | <.0001 | | | |  |
| KiGGS age group | 6-9 years | REF | |  | KiGGS age group | | 18-31 years | | | REF | | |  | | | |  |
|  | 14-17 years | 0.3618  (-0.1962-0.9198) | | 0.2037 |  | | 10-13 years | | | -1.2558  (-3.4971-0.9855) | | | 0.272 | | | |  |
|  | 10-13 years | 0.2749  (-0.1714-0.7212) | | 0.2273 |  | | 14-17 years | | | -0.6196  (-2.8846-1.6454) | | | 0.5917 | | | |  |
| CASMIN - Educational groups (maximum of parents in the household) | Intermediate education | REF | |  | CASMIN - Educational groups (maximum of parents in the household) | | Intermediate education | | | REF | | |  | | | |  |
|  | Basic education | 2.4539  (0.9425-3.9654) | | 0.0015 |  | | Basic education | | | -0.3275  (-0.9253-0.2704) | | | 0.2828 | | | |  |
|  | Higher education | 0.2953  (-0.0655-0.6561) | | 0.1087 |  | | Higher education | | | 0.1526  (-0.2759-0.5811) | | | 0.4849 | | | |  |
| At least one Meningococcal C vaccination | Unvaccinated | REF | |  | Allergies: Biological parents (self-report) | | No | | | REF | | |  | | | |  |
|  | Vaccinated (at least one vaccination) | 0.6295  (0.0873-1.1717) | | 0.0229 |  | | Yes | | | 0.453  (0.1619-0.7441) | | | 0.0023 | | | |  |
| Bronchial asthma  (12-month prevalence) | No | REF | |  | Birch (Bet v 1; t215; PR-10 protein) recombinant sensitization | | No (CAP class = 0) | | | REF | | |  | | | |  |
|  | Yes | -0.8658  (-1.4716-0.2599) | | 0.0051 |  | | Yes (CAP class >=1) | | | 1.4553  (0.6951-2.2155) | | | 0.0002 | | | |  |
| General health status  (in 3 categories) | Very good | REF | |  | Constantly fidgety | | Partially applies | | | REF | | |  | | | |  |
|  | Good | 0.4456  (0.166-0.7253) | | 0.0018 |  | | Does not apply | | | 0.7703  (0.3481-1.1925) | | | 0.0004 | | | |  |
|  | Average/poor/very poor | -0.1918  (-0.8535-0.4699) | | 0.5699 |  | | Definitely applies | | | -0.305  (-1.1126-0.5027) | | | 0.459 | | | |  |
| Overweight (>P90) | No | REF | |  | Contact with farm or stable animals at ages 0-6 years | | No | | | REF | | |  | | | |  |
|  | Yes | -0.5541  (-1.1716-0.0634) | | 0.0786 |  | | Yes | | | -0.3961  (-0.7508-0.0414) | | | 0.0287 | | | |  |
| Personal belongings of the child: Laptop | Yes | REF | |  | Difficulties: Impairment with friends | | Difficult | | | REF | | |  | | | |  |
|  | No | -0.9786  (-1.935--0.0221) | | 0.0449 |  | | Clearly | | | 0.8458  (-1.1092-2.8007) | | | 0.3963 | | | |  |
|  | not asked | -7.1521  (-17.3132-3.009) | | 0.1676 |  | | Hardly | | | 0.7357  (-1.1024-2.5738) | | | 0.4326 | | | |  |
| Personal belongings: PC | Yes | REF | |  |  | | Not at all | | | -0.3457  (-2.1916-1.5002) | | | 0.7135 | | | |  |
|  | No | 0.4369  (0.059-0.8149) | | 0.0235 | Efforts for health-related activities in the environment | | Quite difficult | | | REF | | |  | | | |  |
| Personal belongings: Smartphone | Yes | REF | |  |  | | Very easy | | | -0.6664  (-1.3013-0.0315) | | | 0.005 | | | |  |
|  | No | 0.4325  (0.0761-0.7889) | | 0.0174 |  | | Quite easy | | | 0.999  (-1.4746-0.5234) | | | 0.0397 | | | |  |
| SDQ Inattention/Hyperactivity, international standardization | Unconspicuous | REF | |  | 5 | | Very difficult | | | 1.4191  (-2.4099-0.4283) | | | <.0001 | | | |  |
|  | Conspicuous | -0.1411  (-1.0879-0.8057) | | 0.7701 | Hazelnut (f17) sensitization | | No (CAP class = 0) | | | REF | | |  | | | |  |
|  | Borderline | 0.1577  (-0.7134-1.0288) | | 0.7226 |  | | Yes (CAP class >=1) | | | -0.938  (-1.6518-0.2242) | | | 0.01 | | | |  |
| SDQ Peer problems scale score |  | 0.0863  (-0.0143-0.1869) | | 0.0926 | Life events: Death of a close person | | No | | | REF | | |  | | | |  |
| SDQ Prosocial behavior, international standardization | Unconspicuous | REF | |  |  | | Yes | | | 0.1034  (-0.2835-0.4903) | | | 0.6001 | | | |  |
|  | Borderline | 0.4381  (-0.0883-0.9646) | | 0.1028 | Life events: Death of a close person X CASMIN - Educational groups | | Yes X Basic education | | | 1.9009  (0.7728-3.029) | | | 0.001 | | | |  |
|  | Conspicuous | 0.13  (-0.6152-0.8752) | | 0.7323 |  | | Yes X Higher education | | | -0.1292  (0.7728-3.029) | | | 0.6729 | | | |  |
| Smoking status (mother) | No | REF | |  | Life events: Own prolonged hospital stay | | No | | | REF | | |  | | | |  |
|  | Yes, occasionally | 0.6518  (-0.2023-1.506) | | 0.1346 |  | | Yes | | | 0.3045  (-0.2724-0.8814) | | | 0.3007 | | | |  |
|  | Yes, daily | 0.1197  (-0.3124-0.5519) | | 0.5870 | Life events: Own prolonged hospital stay X CASMIN - Educational groups | | Yes X Higher education | | | 0.2033  (-0.7046-1.1130) | | | 0.6613 | | | |  |
| Stealing at home/school/elsewhere | Partially applies | REF | |  |  | | Yes X Basic education | | | -2.8068  (-4.1996-1.4139) | | | <.0001 | | | |  |
|  | Definitely applies | 3.2494  (1.111-5.3878) | | 0.0029 | Life events: Own serious accident | | No | | | REF | | |  | | | |  |
|  | Does not apply | 1.1688  (0.4727-1.8649) | | 0.0010 |  | | Yes | | | -0.8308  (-1.404-0.2576) | | | 0.0045 | | | |  |
| Surgery for removal of polyps | No | REF | |  | Parental behavior towards the child: Desired changes in the child | | Applies fairly | | | REF | | |  | | | |  |
|  | Yes | 0.3769  (0.0517-0.7021) | | 0.0231 |  | | Applies slightly | | | -1.1075  (-1.6816-0.5334) | | | 0.0002 | | | |  |
| Type of participation | Examination and Survey | REF | |  |  | | | Applies completely | | | -1.1206  (-2.127-0.1143) | | | 0.0291 | | | |
|  | Survey | 31.4568  (8.148-54.7656) | | 0.0082 |  | | | Does not apply | | | -1.1413  (-1.7422-0.5404) | | | 0.0002 | | | |
| Type of Questionnaire | 3-10 years cross-section | REF | |  | Parental behavior towards the child: Encouraging after poor grades | | | Applies fairly | | | REF | | |  | | | |
|  | 11-17 years cross-section | -2.5176  (-4.0995- -0.9356) | | 0.0018 |  | | | Does not apply | | | 1.3894  (-0.0476-2.8265) | | | 0.0581 | | | |
| Varicella: first vaccination by the end of the 24th month of life | No vaccination by the end of the 2nd year of life | REF | |  |  | | | Applies completely | | | 0.4956  (0.1937-0.7974) | | | 0.0013 | | | |
|  | First vaccination by the end of the 2nd year of life | -0.6789  (-1.1842-0.1736) | | 0.0085 |  | | | Applies slightly | | | 0.7913  (0.2325-1.3501) | | | 0.0055 | | | |
| Z-score for mean systolic blood pressure (KiGGS as reference) |  | 0.3226  (0.0826-0.5626) | | 0.0084 | Participation type | | | Examination and survey | | | REF | | |  | | | |
| ISCED Educational groups (maximum of parents in the household) | Higher education | REF | |  |  | | | Survey | | | 0.0651  (-0.3185-0.4487) | | | 0.7392 | | | |
|  | Basic education | -1.2068  (-1.9107-0.5029) | | 0.0008 | Quality of life: Physical activity | | | Not at all | | | REF | | |  | | | |
| SDQ Inattention/Hyperactivity, international standardization X gender | Borderline X male | -1.3757  (-2.532-0.2193) | | 0.0197 |  | | | Fairly | | | 1.0002  (0.1403-1.8601) | | | 0.0226 | | | |
|  | Conspicuous X male | -0.4144  (-1.5557-0.7269) | | 0.0042 |  | | | A little | | | 0.3102  (-0.5847-1.2051) | | | 0.4967 | | | |
| Smoking status (mother) X CASMIN - Educational groups | Yes, occasionally X Basic education | -4.0061  (-6.7454-1.2668) | | 0.0042 |  | | | Moderately | | | 0.1987  (-0.662-1.0593) | | | 0.6508 | | | |
|  | Yes, occasionally X Higher education | -1.7855  (-6.7454-1.2668) | | 0.0042 |  | | | Very | | | 0.1506  (-0.7071-1.0083) | | | 0.7306 | | | |
|  | Yes, daily X Higher education | -0.3541  (-1.638-0.2086) | | 0.3976 | Residence status of parents | | | Permanent | | | REF | | |  | | | |
|  | Yes, daily X Basic education | -0.7147  (-1.638-0.2086) | | 0.1292 |  | | | German or EU citizen | | | 0.8714  (0.2744-1.4685) | | | 0.0042 | | | |
|  |  |  | |  | Smoking yes/no (mother) | | | No | | | REF | | |  | | | |
|  |  |  | |  |  | | | Yes | | | -0.5585  (-0.9241-0.193) | | | 0.0028 | | | |

^1^ CI = confidence interval
